# Virological outcomes of third-line antiretroviral therapy in a global context: a systematic reviews and meta-analysis

**DOI:** 10.1186/s12981-024-00630-7

**Published:** 2024-06-25

**Authors:** Tegene Atamenta kitaw, Biruk Beletew Abate, Gizachew Yilak, Befkad Derese Tilahun, Abebe Merchaw Faris, Getachew Tesfaw Walle, Ribka Nigatu Haile

**Affiliations:** Department of Nursing, College of Health Science, Woldia University, Woldia, Ethiopia

**Keywords:** Virological outcomes, Third-line antiretroviral therapy, Global, Review

## Abstract

**Background:**

Despite remarkable progress, HIV’s influence on global health remains firm, demanding continued attention. Understanding the effectiveness of third-line antiretroviral therapy in individuals who do not respond to second-line drugs is crucial for improving treatment strategies. The virological outcomes of third-line antiretroviral therapy vary from study to study, highlighting the need for robust global estimates.

**Methods:**

A comprehensive search of databases including PubMed, MEDLINE, International Scientific Indexing, Web of Science, and Google Scholar, was conducted. STATA version 17 statistical software was used for analysis. A random-effects model was applied to compute the pooled estimates. Subgroup analysis, heterogeneity, publication bias, and sensitivity analysis were also performed. The prediction interval is computed to estimate the interval in which a future study will fall. The GRADE tool was also used to determine the quality of the evidence.

**Results:**

In this systematic review and meta-analysis, 15 studies involving 1768 HIV patients receiving third-line antiretroviral therapy were included. The pooled viral suppression of third-line antiretroviral therapy was 76.6% (95% CI: 71.5- 81.7%). The viral suppression rates at 6 and 12 months were 75.5% and 78.6%, respectively. Furthermore, third-line therapy effectively suppressed viral RNA copy numbers to ≤ 50 copies/mL, ≤ 200 copies/mL, and ≤ 400 copies/mL with rates of 70.7%, 85.4%, and 85.7%, respectively.

**Conclusion:**

More than three-fourths of patients on third-line antiretroviral therapy achieve viral suppression. Consequently, improving access to and timely initiation of third-line therapy may positively impact the quality of life for those with second-line treatment failure.

**Supplementary Information:**

The online version contains supplementary material available at 10.1186/s12981-024-00630-7.

## Key Summary Points

• Understanding the effectiveness of third-line antiretroviral therapy in individuals who do not respond to second-line drugs is crucial for improving treatment strategies.
• Currently, second-line treatment failure has been increasing, making progression to third-line therapy seemingly mandatory in many settings, making it apparent that recent evidence is crucial.
• Viral suppression rate of third-line ART was 76.6% (95% CI: 71.5- 81.7%).
• The viral suppression rates at 6 and 12 months were 75.5% and 78.6%.
• Third-line therapy effectively suppressed viral RNA copy numbers to ≤ 50 copies/mL, ≤ 200 copies/mL, and ≤ 400 copies/mL with rates of 70.7%, 85.4%, and 85.7%, respectively.

**Supplementary Information:**

The online version contains supplementary material available at 10.1186/s12981-024-00630-7.

## Introduction

Despite significant progress, HIV remains a pressing public health issue worldwide. At the end of 2022, more than 39 million people were infected with the virus, and ongoing transmission of the virus continued in every country. Worryingly, some regions that had previously experienced declines in new infections are now experiencing increases [1]. Africa is disproportionately affected by the epidemic, with the WHO (World Health Organization) African Region accounting for two-thirds of all cases. In 2022, 630,000 individuals died from HIV-related causes, and 1.3 million new infections emerged [2]. Nearly 67% of all HIV cases globally were from sub-Saharan Africa (SSA) [3].

Antiretroviral drugs have played a crucial role in substantially decreasing the transmission of HIV on a global scale [3]. Despite their positive impact, concerns have emerged due to the rising instances of drug resistance and the challenges associated with achieving virological suppression [4]. The increasing resistance to these drugs and the failure to achieve effective suppression of the virus have become significant issues, posing potential obstacles to ongoing efforts to combat HIV transmission and improve overall public health [5].

The WHO recommends the use of third-line ART after the failure of second-line treatment [6]. Salvage regimens for third-line ART, which involve the use of medications such as darunavir, raltegravir, and etravirine, have previously demonstrated satisfactory rates of virologic suppression in clinical trials [7, 8]. Despite the WHO advising that all countries make available ART, only a few countries can offer these treatment regimens because of the considerable expenses and challenges associated with their implementation [9].

Achieving and maintaining virological suppression is crucial for HIV patients, as it prevents transmission, slows disease progression, and improves overall health. Studying third-line ART outcomes helps assess the effectiveness of ART in suppressing viral replication in patients who have failed previous regimens. Understanding virological outcomes across different regions and populations informs the development of optimal third-line ART regimens tailored to specific needs and resistance patterns. While HIV disproportionately affects individuals in low- and middle-income countries, this research will inform public health policies and resource allocation to ensure equitable access to effective third-line ART globally. Comprehensive data on third-line ART outcomes are lacking in many regions, hindering informed decision-making and creating knowledge gaps that this study can help address. Overall, studying virological outcomes of third-line ART in a global context is essential for improving patient care, optimizing treatment strategies, and achieving equitable access to effective HIV therapy.

### Objectives

To assess the pooled estimate of virological outcomes of third-line ART in a global context.

To determine the virological outcomes of third-line ART at different levels.

## Methods

### Protocol development and registration

This review was designed in accordance with preferred methods of reviewing available Systematic Review and Meta-analysis (SRM) studies and the Strengthening the Reporting of Observational Studies in Epidemiology (STROBE) guidelines [10, 11]. First, a similar review was checked on PROSPERO, and no similar studies were found. The protocol of this review was subsequently summarized and registered as CRD42024499263 in PROSPERO. PROSPERO registration -related information is available upon reasonable request from the primary author. This systematic review and meta-analysis focused on a systematic synthesis of existing studies on the virological outcomes of third-line ART in a global context.

### Search strategy and information sources

A comprehensive literature search was conducted for studies reported virological outcomes of third-line ART in the Embase, Web of Science, PubMed, Scopus, International Scientific Indexing (ISI), and Google Scholar databases. using the PICO frameworks. Combinations, keywords and MeSH terms were used to retrieve the studies. In addition, the snowballing technique was used to retrieve additional studies from the citation lists of the articles found in the available databases. Gray literature and manual searches were also performed to find unindexed/not published/researched articles. The search strategies were drafted using concepts and key search terms. The first concept: virological outcome: “treatment outcome”, “treatment responses”, “effectiveness”, “virological suppression”, and “outcome”. The second concept included the following: “third-line regimen”, “third-line therapy”, “third-line antiretroviral therapy”, “third-line highly active antiretroviral therapy”, and “third-line HAART”. Literature searches were independently conducted by two authors (TAK and RNH). Any inconsistency was resolved by agreement. In the case of articles with incomplete information, the primary authors of the respective article were contacted. We used the search terms “OR” or “AND” independently and/or in combination. In addition, ‘related article’ and ‘citied by’ features of the PubMed database were used to find articles from the included studies.

### Eligibility criteria

#### Inclusion criteria

A study reporting the virological outcome of third line ART written in the English language was included. For respective study to be considered for this systematic review and meta-analysis, it should fulfill the following prioritized criteria. Condition: The outcome of interest should be measured as the virological outcome of third line ART. Context: Setting can be anywhere. Cross-sectional studies and cohort studies (prospective and retrospective) were eligible. Population: HIV-1 infected individuals who were receiving third line ART. All population group can be included without age restrictions.

### Exclusion criteria and definitions

Articles were excluded for one of the following reasons: (1) did not measure the outcome of interest for this systematic review and meta-analysis, (2) were written in languages other than English; or (3) were narrative reviews, expert opinions, case reports, editorials, correspondences, abstracts, or methodological studies.

### Data extraction and management

Two authors (BBA and RNH) conducted the data extraction independently using a standardized extraction form. The title and abstract were first screened and selected, after which the full texts were reviewed. In the case of disagreement, discussion with other reviewers was performed to determine the final selection of articles to include in this review. After the systematic search was complete, potentially eligible articles were imported to EndNote 21. Duplicated studies were removed if two or more articles had common characteristics. The structured data were extracted in the form of a Microsoft Excel spreadsheet. The extracted data included the following: (1) study identification (last name of the primary author and year of publication), (2) setting, (3) sample size, (4) study design, (5) setting, (6) age range of the participant, (7) virological suppression, (8) cutoff point for virological suppression (viral load (VL) copies/ml), and (9) time at which virological suppression was detected. In addition, the percentage of virological suppression from each study was computed using the number of participants declaring suppressed viral RNA/ml as the numerator and the total number of sample sizes as the denominator. The corresponding author was contacted when any difficulties were encountered during data extraction.

### Risk of bias assessment

The Joanna Briggs Institute (JBI) critical appraisal tool provided a measure of methodological quality for this systematic review and meta-analysis. Two independent reviewers evaluated each study using a series of “Yes,” “No,” or “Unclear” questions. To ensure objectivity, any disagreements were resolved through consensus among the authors and an independent reviewer. A scoring system (1 for “Yes,” 0 for “No,” U for “Unclear”) was applied, with final scores converted into percentages for risk-of-bias ranking: ≤49% (high), 50–69% (moderate), and above 70% (low). Only studies scoring at least 50% (indicating moderate or low risk of bias) were included. For ongoing reviewer disputes, individual ratings were averaged. The quality of each primary study’s results was documented in a dedicated column within the data extraction form to facilitate further analysis.

### Statistical analysis

Once the data extraction was completed in Microsoft Excel, the data were imported to STATA version 17 software for analysis. Qualitative and narrative methods were employed to summarize the estimates of the included studies. When two or more estimates on the same topic were found, the range of the estimate and/or pooled estimate was used. The standard error was computed by considering a binomial distribution formula. The overall virological outcome (suppression) was pooled using a random effects model [12]. In addition, the pooled estimates were presented by using a forest plot. Cochrane’s Q statistics (chi-square), inverse variance (I^2^) and p-values [13] were computed to show the level of heterogeneity between studies. Zero invers variance (I^2^) revealed true homogeneity, whereas 25%, 50% and 75% had low, moderate and high heterogeneity, respectively [14, 15]. Subgroup analysis was performed according to publication year, age of the participant, study quality (JBI quality score), country income level, duration of viral load suppression and number of viral RNA copies. Leave one out (sensitivity) meta-analysis was performed to determine the effect of a single study on the overall pooled estimation. A funnel plot was constructed, and Egger’s regression test was used to determine publication bias [16].

### Prediction interval

A prediction interval was computed to estimate how much variation we can expect in the results of a new study if that study was randomly chosen from the same group of studies used in the current analysis. This interval helps us understand how much the combined result might vary depending on the specific new study included [17].

### Assessment of the quality of evidence

We employed a powerful tool called the GRADE tool (Grading of Recommendations Assessment, Development and Evaluation) to check how confident the pooled estimate is. Evidence was **assessed** based on five main domains (risk of bias, consistency, directness, precision, and publication bias) [18]. (Table 1).

**Table 1:** GRADE Quality of Evidence Interpretations

Grade	Definition
High	Further research is very unlikely to change our confidence in the estimate of effect.
Moderate	Further research is likely to have an important impact on our confidence in the estimate of effect and may change the estimate.
Low	Further research is very likely to have an important impact on our confidence in the estimate of effect and is likely to change the estimate.
Very low	Any estimate of effect is very uncertain

## Results

**Fig. 1:**
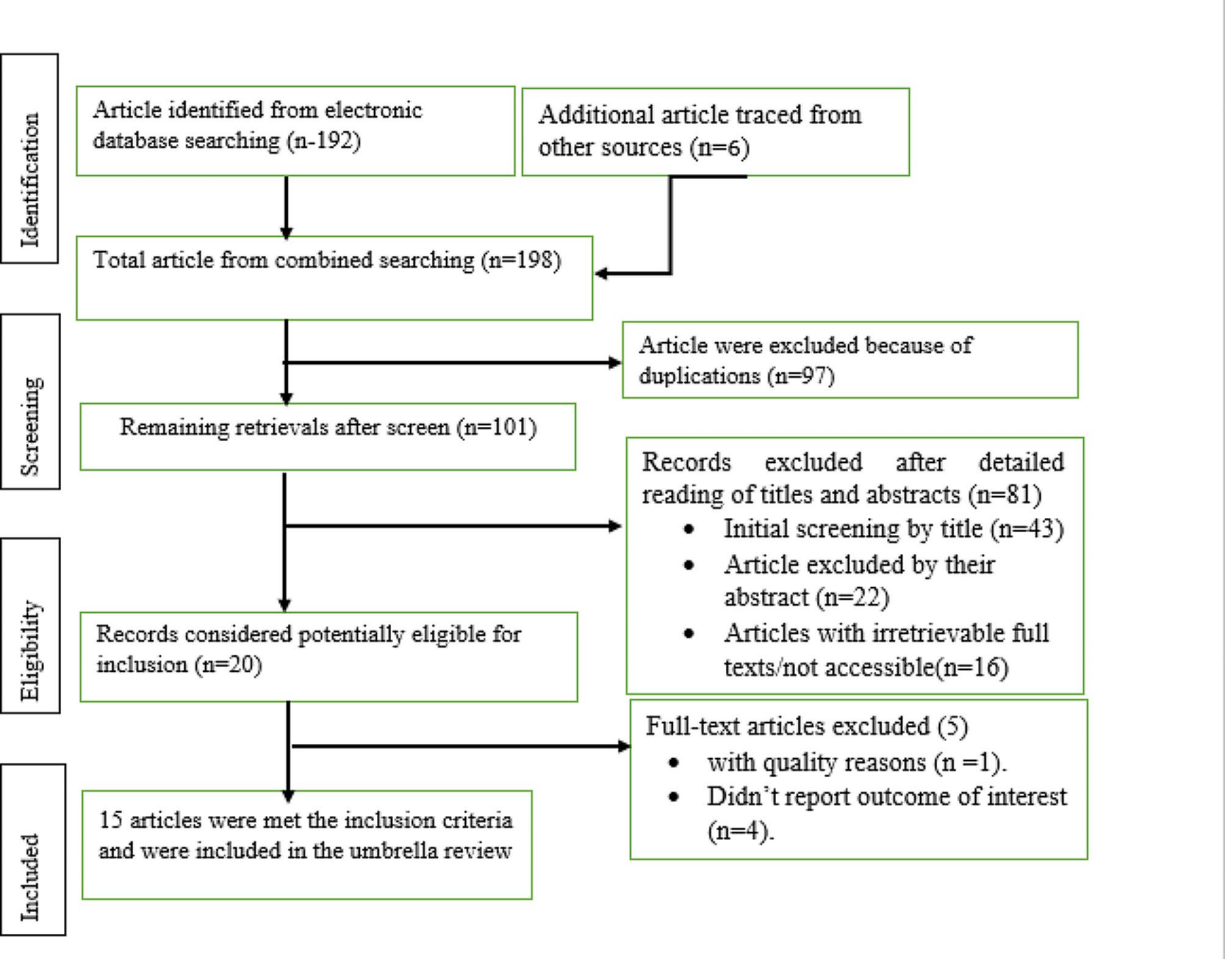
Flow chart diagram describing the selection of studies for systematic review and meta-analysis of virological outcomes of third-line antiretroviral therapy in a global context, 2024

A total of 198 records were retrieved from different database search engines. Ninety-seven of them were excluded because of duplications through the EndNote citation manager. From 101 records, 81 retrievals were excluded after detailed reading of the titles and abstracts. The remaining 20 records were potentially eligible for inclusion. After thoroughly checking the full publications of 20 articles, 5 studies were removed because of quality concerns and because their outcome estimates varied from the outcome of interest. Finally,15 eligible studies [19–33] were included in this systematic review and meta-analysis to estimate virological outcomes of third-line ART in a global context. (Fig. 1).

### Characteristics of the original studies

All included studies were published from 2017 onward and included only primary studies that were published from 2015 to 2023. In this systematic review and meta-analysis, a total of 1768 HIV patients receiving third-line ART were included to estimate the virological outcome of the treatment. Most of the studies (10) were from the African continent. Thirteen studies were cohort studies. (Table 2).

**Table 2:** Summary of the key characteristics of the studies included in this systematic review and meta-analysis, 2024

Authorsname	Year	Study setting	Study design	Age group included	Sample size	Virological suppressed	JBI(%)
(Andronescu et al., 2019)	2019	Zambia	Retrospective cohort	All age group	80	50	72.73
(Avihingsanon et al., 2022)	2022	Low- and middle-income	Cohort	All age group	257	224	81.82
(Chakravarty et al., 2023)	2023	India	Cohort	All age group	72	59	72.73
(Chimbetete et al., 2018)	2018	Zimbabwe	Cohort	All age group	36	29	81.82
(Chimbetete et al., 2020)	2020	Zimbabwe	Cohort	All age group	111	83	63.64
(Evans et al., 2018)	2018	South Africa	Retrospective	≥18 years	42	35	90.91
(Meintjes et al., 2015)	2015	Southern African	Retrospective cohort	≥18 years	152	126	81.82
(Moorhouse et al., 2019)	2019	South African	Cohort	All age group	118	93	90.91
(Nuttall and Pillay, 2018)	2018	South Africa	Cross-sectional	<18 year	30	29	72.73
(Prasitsuebsai et al., 2017)	2017	Thailand	Cross-sectional	<18 year	50	33	72.73
(Subramanian et al., 2021)	2021	India	Cohort	All age group	232	151	81.82
(Toeque et al., 2022)	2022	Zambia	Cohort	All age group	345	225	90.91
(Zulu et al., 2021)	2021	Zambia	Retrospective cohort	≥18 years	66	48	72.73
(EPPICC et al., 2022)	2022	Europe and Thailand	Cohort	<18 year	141	97	81.82
(Tiam et al., 2020)	2020	Sub-Saharan Africa	Cohort	<18 year	36	29	72.73

### Quality of the included studies

Our systematic review and meta-analysis prioritized rigorous methodology, applying the Joanna Briggs Institute’s (JBI) critical appraisal tool to meticulously assess the quality of the included cross-sectional and cohort studies. Thus, the lowest percentage of quality assessment was 63.6%. A closer look revealed that only one study was identified as having a moderate risk of bias based on the JBI assessment.

### Virological outcomes of third-line antiretroviral therapy

**Fig. 2:**
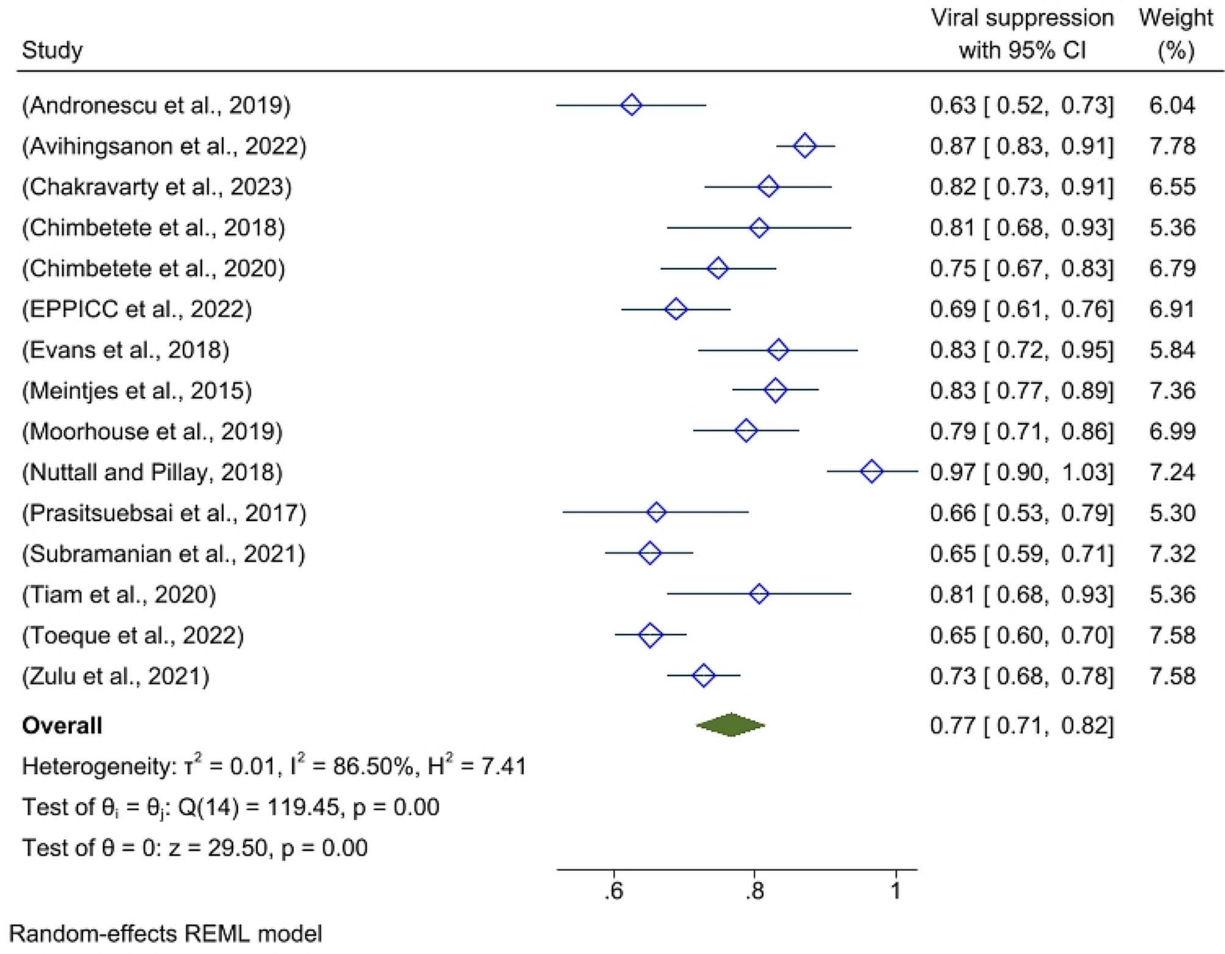
Global pooled viral suppression of third-line antiretroviral therapy in the global context, 2024

This meta-analysis identified heterogeneity across the studies (I^2^ = 86.5%, p-value < 0.001, H^2^ = 7.41 and T^2^ = 0.001). As a result, we used a random effects model to estimate the pooled viral suppression effect of third-line ART. The results of 15 studies revealed that the pooled viral suppression of third-line ART was 76.6% (95% CI: 71.5- 81.7%). (Fig. 2).

### Prediction interval

A prediction interval tells us how much variation we can expect in the results of a new study if that study were randomly chosen from the same group of studies used in the current analysis. The table shows how much the combined result might vary depending on the specific new study included [17]. According to the results of this systematic review and meta-analysis, the prediction interval for pooled viral suppression following third-line ART was 0.562 and 0.970, respectively. Thus, if we add a new study, the effect size will fall within the above ranges.

### Publication bias

Substantial publication bias was assessed objectively using both Begg’s and Egger’s tests. Neither the Begg’s nor Egger’s test revealed publication bias with p- values of 0.9618 and 0.7215, respectively. Moreover, a symmetrical distribution of funnel plots was also found.

### Subgroup analysis

**Fig. 3:**
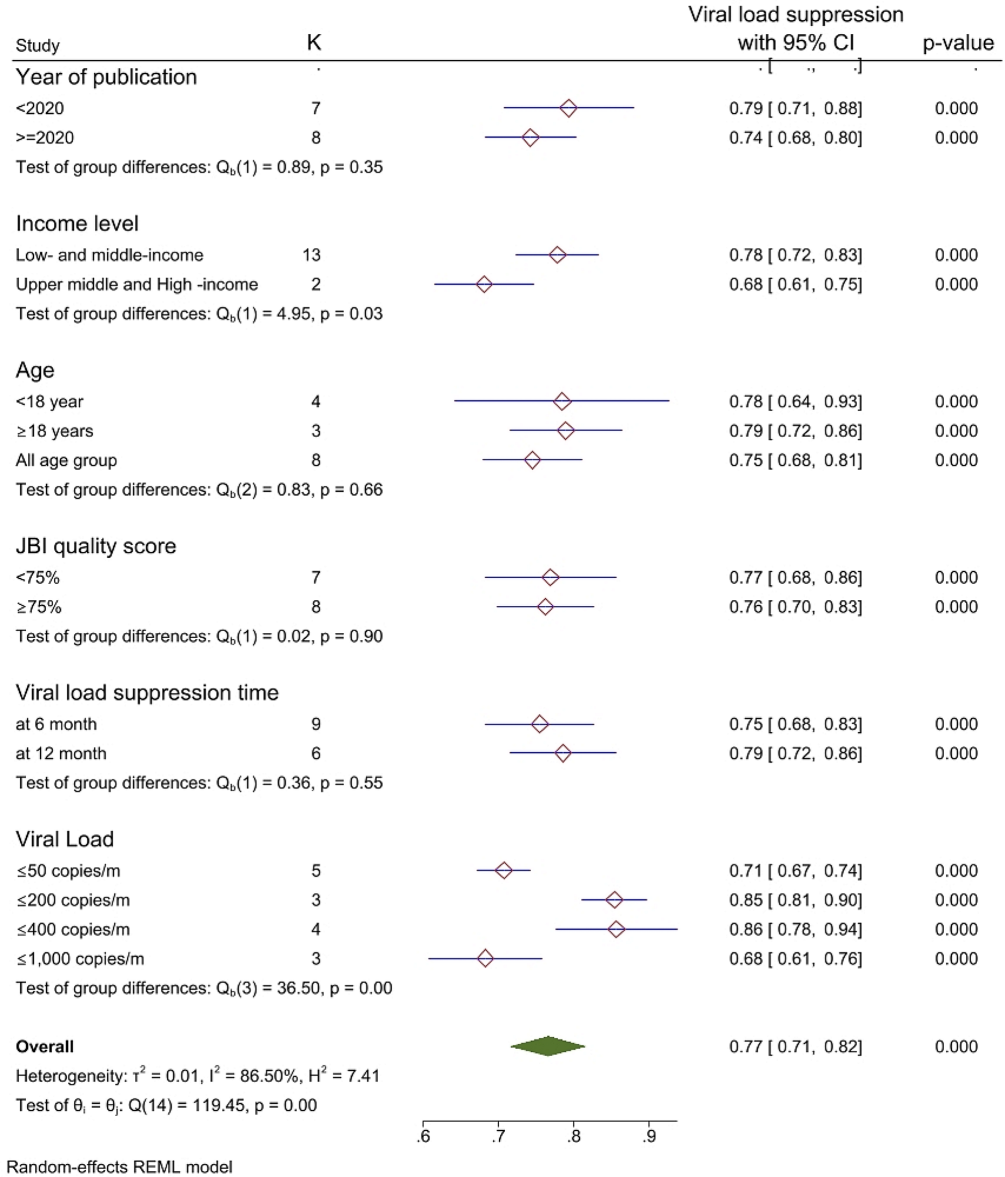
Subgroup analysis of the virological outcomes of third-line antiretroviral therapy in the global context, 2024

Subgroup analysis was performed according to publication year, age of the participant, study quality (JBI quality score), country income level, duration of viral load suppression and number of viral RNA copies. Thus, in low- and middle-income countries, 77.8% (95% CI: 72.3–83.3) of patients achieved viral suppression after switching to third-line therapy. Additionally, the viral suppression rates at 6 and 12 months were 75.5% and 78.6%, respectively. Furthermore, the analysis revealed that third-line therapy effectively suppressed viral RNA copies to ≤ 50 copies/mL, ≤ 200 copies/mL, and ≤ 400 copies/mL, for a total of 70.7%, 85.4%, and 85.7%, respectively. No significant difference in viral suppression was observed between participants older than and younger than 18 years (78.4% vs. 78.9%). (Fig. 3).

### Sensitivity analysis

Leave-one-out analysis was also conducted to explore the influence of a single study on the overall effect size estimate. A leave-one-out meta-analysis omits the corresponding study, and a meta-analysis is performed on the remaining studies (*n* = 1). If the cross-ponding study confidence interval does not include the overall effect size estimate (theta), it is declared that the study significantly influences the overall effect size estimate [34]. In this study, the general effect size estimate (theta) was 76.6 and was included within the confidence intervals of all the studies. Thus, omitting one study did not significantly influence the overall effect size estimate. (Table 3).

**Table 3:** Leave one out meta-analysis to explore the influence of one study on the overall pooled virological outcomes of third-line antiretroviral therapy in a global context, 2024

Omitted study	theta	[95% conf. interval]	*p* value
(Andronescu et al., 2019)	0.775	[0.724–0.826]	< 0.001
(Avihingsanon et al., 2022)	0.757	[0.705–0.809]	< 0.001
(Chakravarty et al., 2023)	0.762	[0.708–0.816]	< 0.001
(Chimbetete et al., 2018)	0.764	[0.710–0.817]	< 0.001
(Chimbetete et al., 2020)	0.767	[0.713–0.822]	< 0.001
(EPPICC et al., 2022)	0.772	[0.718–0.825]	< 0.001
(Evans et al., 2018)	0.762	[0.708–0.815]	< 0.001
(Meintjes et al., 2015)	0.761	[0.707–0.815]	< 0.001
(Moorhouse et al., 2019)	0.764	[0.709–0.819]	< 0.001
(Nuttall and Pillay, 2018)	0.750	[0.706–0.795]	< 0.001
(Prasitsuebsai et al., 2017)	0.772	[0.719–0.824]	< 0.001
(Subramanian et al., 2021)	0.775	[0.723–0.827]	< 0.001
(Tiam et al., 2020)	0.764	[0.710–0.817]	< 0.001
(Toeque et al., 2022)	0.775	[0.724–0.827]	< 0.001
(Zulu et al., 2021)	0.769	[0.714–0.824]	< 0.001
theta	0.766	[0.715–0.817]	< 0.001

### GRADE quality evidence assessment

We employed the Grading of Recommendations Assessment, Development and Evaluation (GRADE) tool, a well-established framework for assessing evidence certainty, to meticulously appraise the strength of evidence for each investigated outcome. While acknowledging that observational studies typically begin as “low quality” evidence in the GRADE system, they downgraded them further to “very low” due to concerns in specific domains. Assessments were made for the five main domains (risk of bias, consistency, directness, precision and publication bias). Thus, this systematic review and meta-analysis has a low quality of evidence Our confidence in the effect estimate is limited: the true effect may be substantially different from the estimate of the effect. Since observational studies were included, the overall evidence was downgraded. The detailed quality of evidence assessment is presented in the supplementary file.

## Discussion

This systematic review and meta-analysis aimed to estimate the global viral outcomes of third-line ART in HIV-infected patients. Our analysis revealed that 76.6% (95% CI: 71.5–81.7%) of patients achieved viral suppression after switching to third-line therapy. The viral suppression rates of third-line therapy were 75.5% and 78.6% at 6 and 12 months, respectively. Notably, in low- and middle-income countries, 77.8% (95% CI: 72.3–83.3) of patients achieved viral suppression with third-line therapy. Furthermore, the analysis demonstrated the efficacy of third-line therapy in suppressing viral RNA copies, achieving rates of 70.7%, 85.4%, and 85.7% for levels ≤ 50 copies/mL, ≤ 200 copies/mL, and ≤ 400 copies/mL, respectively.

This systematic review and meta-analysis revealed that, 70.7% of the patients receiving third-line therapy achieved an undetectable viral load (≤ 50 copies/mL), as defined by reference [35]. A reduced viral load minimizes the risk of AIDS-related complications and opportunistic infections, leading to improved quality of life and a longer lifespan. An undetectable viral load significantly reduces the risk of transmitting HIV to sexual partners, contributing to reduced community transmission and improved public health. The potential of third-line therapy to reach marginalized communities and individuals who previously lacked access to effective treatment options due to geographic, economic, or social barriers should be emphasized.

A total of 85.7% of patients achieved viral suppression at ≤ 400 copies/mL. These findings are in agreement with those of a study performed in Africa, in which 71.1% of the patients had ≤ 50 copies/ml and 82.9% had ≤ 400 copies/ml [25]. The similarity between the findings and those from the study in Africa adds to the generalizability of these results. These findings suggest that the high viral suppression rates observed are not limited to a specific setting but rather more broadly reflect the potential of third-line ART in LMICs. This finding strengthens the need for widespread access to effective third-line therapy in resource-limited regions where second -line therapy failure is high. Achieving an undetectable viral load (< 50 copies/mL) is crucial for preventing further HIV transmission and disease progression. These high suppression rates suggest that third-line ARTs effectively achieve this goal, leading to improved individual and public health outcomes.

Furthermore, the rates of viral suppression in patients receiving third-line therapy were 75.5% and 78.6% at 6 and 12 months, respectively. Achieving viral suppression in more than 75% of patients at both 6 and 12 months of age suggests that third-line therapy is a highly effective option for managing HIV after failed first- and second-line regimens, thereby improving quality of life. Even at low levels, continued viral replication can lead to resistance against existing medications [36]. Monitoring and managing resistance are crucial for sustaining the success of third-line therapy. Third-line therapies can be expensive [37], and ensuring equitable access for all patients, particularly in resource-limited settings, remains a challenge.

This systematic review and meta-analysis build upon a robust observational study encompassing global research on virological outcomes of third-line ART. This approach offers a comprehensive understanding of the virological outcomes of third-line ART. The research methodology adhered to the rigorous PRISMA guidelines, guaranteeing the inclusion of high-quality and relevant studies. In addition, we evaluated the overall evidence of the findings with the GRADE tool. Furthermore, this review employs the JBI tool to rigorously assess the methodological quality of each included study, providing an additional layer of confidence in the findings. Despite its strengths, the study has several limitations. Although the study aimed for a global context, the included studies did not represent all the world regions. This geographical bias limits the generalizability of the pooled results. Finally, the high level of heterogeneity among the included studies is another limitation. While these findings are promising, longer-term follow-up studies are crucial for assessing the durability of viral suppression and identifying potential late-stage treatment failure or resistance. The long-term safety and side effects of third-line therapies require further investigation, especially for patients with multiple prior treatment lines.

## Conclusions

This study found that three-fourths of patients who switched to third-line regimens achieved viral suppression. Additionally, 70.7% of those on third-line therapy reached an undetectable viral load (≤ 50 copies/mL), reducing transmission risk. These findings are particularly relevant in low- and middle-income countries, where treatment options may be limited. This highlights the potential of these regimens to improve health outcomes for vulnerable populations. Given these success rates, it is important to consider healthcare policies that enhance access to third-line therapies for patients who do not respond to first- and second-line treatments. Increasing access to third-line therapies is therefore advisable. We suggest that before increasing access to third-line therapy management, particularly in low- and middle-income countries, careful consideration must be given to the safety and side effects of these regimens. It is essential to assess whether people living with HIV can tolerate potential adverse effects, especially given existing challenges such as socio-economic problems and poverty. Therefore, we recommend conducting comprehensive evaluations of the safety profile of third-line therapies in the context of local healthcare settings and patient populations. Additionally, strategies to mitigate potential side effects and support adherence should be integrated into HIV treatment programs to ensure optimal outcomes for individuals receiving these regimens.

### Recommendations and future research outlook

Updating official guidelines to include third-line options as a legitimate and effective course of action for treatment failure thereby empowers healthcare providers to make informed decisions for their patients. Implementing insurance coverage mechanisms: Expanding insurance coverage to include third-line therapies helps alleviate the financial burden on patients and ensures that they can access the treatments they need. Supporting medication affordability programs: Government initiatives, public-private partnerships, and nonprofit organizations can play a crucial role in negotiating lower drug prices and implementing programs that subsidize or offer free third-line medications to patients in need.

In addition to these policy changes, ongoing research is also crucial for optimizing third-line treatment strategies. This includes investigating new drug combinations and therapeutic regimens for improved efficacy and reduced side effects. Strategies for addressing adherence challenges are lacking, as adherence remains a critical factor in achieving and maintaining viral suppression. Monitoring and managing drug resistance, such as continued viral replication even at low levels can lead to resistance against existing medications. In addition, a long-term prospective study is recommended for evaluating the long-term outcomes and sustainability of third-line ART.

### Electronic supplementary material

Below is the link to the electronic supplementary material.

Supplementary Material 1

## Data Availability

No datasets were generated or analysed during the current study.
