# Supplementary material for: Virological outcomes of third-line antiretroviral therapy in a global context: a systematic reviews and meta-analysis
Source: AIDS Res Ther. 2024 Jun 25;21:43. doi: 10.1186/s12981-024-00630-7 (PMC11197289; doi:10.1186/s12981-024-00630-7)
Supplement: Supplementary file 1 — Supplementary Material 1 [file 12981_2024_630_MOESM1_ESM.pdf]

Table 1:GRADE quality of evidence assessment for systematic review and meta-analysis Virological outcomes of third-line antiretroviral therapy in global context, 2024.

GRADE Domain	***Relevant content***	***Explanation***
Risk of bias	*No serious risk of bias, (do not downgrade)*	*The lowest percentage of quality assessment found to be 63.6% (one study only).*
Imprecision	*No serious imprecision (do not downgrade)*	*The total number of events is greater than 300 (n=1311) and the total number of participants in the review is greater than the number of participants required for a single study.*
Inconsistency	*Some inconsistency exists (downgrade one level)*	*There is a heterogeneity (I^2 = 86.5%, p-value <0.001, H^2=7.41 and T^2=0.001).*
Indirectness	*Some inconsistency indirectness exists (downgrade one level)*	*Population= particular participants or settings are not restricted, outcomes= The outcome of measure is informative (viral RNA copies/ml) (it does not include surrogate measurement).*
Publication bias	*Undetected*	*Begg's and Egger's tests revealed no publication bias at p-values of 0.9618 and 0.7215, respectively.*
Overall quality of evidence		***Low quality of evidence:*** *Our confidence in the effect estimate is limited: the true effect may be substantially different from the estimate of the effect. Since observational studies are included, the overall evidence is downgraded.*

We employed the GRADE approach (Grading of Recommendations Assessment, Development and Evaluation) tool, a well-established framework for assessing evidence certainty, to meticulously appraise the strength of evidence for each investigated outcome. While acknowledging that observational studies typically begin as "low quality" evidence in the GRADE system, they downgraded them

further to "very low" due to concerns in specific domains. Assessments were made for the five main domains (risk of bias, consistency, directness, precision and publication bias).

Risk of bias is limitations in the study design and implementation may bias the estimates of an intervention effect. Most information is from studies at low risk of bias: No serious limitations, No downgrade. The proportion of information from studies at high risk of bias is sufficient to affect the interpretation of results: Serious limitations, downgraded one level.

Inconsistency refers to an unexplained heterogeneity of results. Statistical heterogeneity refers to differences in the effects of interventions and arises because of clinical and/or methodological differences between studies. Consider how much variability there is in the results of studies contributing to the outcome of the study. statistical criteria including tests of heterogeneity (e.g. chi-squared or Chi^2) have a low p-value ($p < 0.05$), I^2 value is large (> 25%). Each outcome was downgraded by one due to inconsistency (tests of heterogeneity $p < 0.05$ and I^2 is large).

Indirectness: the question being addressed by the authors of a systematic review is different from the available evidence regarding the population, intervention, comparator, or an outcome. The quality of the evidence may be downgraded when substitute measurements or surrogate endpoints are measured instead of patient-important outcomes. Might be indirect if included studies were limited to particular participants or settings. not at all (indirectness does not appear to be an issue), one point (some indirectness exists), or two points (indirectness is severe, or there is indirectness from several sources).

Imprecision: results are imprecise when studies include relatively few patients and few events and thus have wide confidence intervals around the estimate of the effect. For dichotomous outcomes, unless events rates are very low (see also point 2 below) information is likely to be insufficient if:• total number of events is less than 300 (a "rule of thumb")• total (cumulative) sample size is lower than the calculated optimal information size: i.e. if the total number of participants in the review is less than the number of

participants required for a single adequately powered trial. not at all (imprecision does not appear to be an issue), one point (some imprecision exists), or two points (very serious imprecision exists).

Publication bias is a systematic under-estimate or an over-estimate of the underlying beneficial or harmful effect due to the selective publication of studies. Funnel plot analysis, Egger weighted regression and Begg rank correlation tests were used to detect publication bias and P-value<0.05 was considered as indicative of statistically significant publication bias. Undetected, do not downgrade and strongly suspected, downgrade one level.

Table 2: GRADE ratings and their interpretation

Symbol	Quality	Interpretation
⨁⨁⨁⨁	**High**	We are very confident that the true effect lies close to that of the estimate of the effect.
⨁⨁⨁O	**Moderate**	We are moderately confident in the effect estimate: the true effect is likely to be close to the estimate of the effect, but there is a possibility that it is substantially different.
⨁⨁OO	**Low**	Our confidence in the effect estimate is limited: the true effect may be substantially different from the estimate of the effect.
⨁OOO	**Very low**	We have very little confidence in the effect estimate: the true effect is likely to be substantially different from the estimate of effect
